# Content and framing analysis of food supplement in the BBC news and evaluation from a nutrition science perspective

**DOI:** 10.3389/fnut.2026.1756606

**Published:** 2026-05-13

**Authors:** Metin Erol, Kubra Feyza Erol, Mustafa Bostancı

**Affiliations:** 1 Department of Social Work, Hamidiye Faculty of Health Sciences, University of Health Sciences, Istanbul, Türkiye; 2 Department of Nutrition and Dietetics, Hamidiye Faculty of Health Sciences, University of Health Sciences, Istanbul, Türkiye; 3 Department of Journalism, Faculty of Communication, Sakarya University, Istanbul, Türkiye

**Keywords:** communication studies, digital communication, content analysis, frame analysis, Wordsmith, dietary supplements, functional food, food supplement

## Abstract

This study aims to identify the framing of dietary supplement news within the context of content analysis and to examine how these frames construct perceptions through specific contexts. For the sample, the international news organization BBC was selected. Using keyword searches on BBC’s official website, news articles were identified, and a simple random sampling method was applied to select the sample. The frames of the sampled news articles were extracted using Wordsmith software. The nutritional dimension of these frames was critically evaluated through eight analytical questions. Within this framework, which types of food supplements are most frequently featured in news content (1), Is there a scientific basis (2), is the benefit or the risk more prominent (3), how does social media shape nutritional behavior (4) and which target group does the news report address (5) The findings indicate that media content predominantly presents supplements in a benefit-oriented manner and positions them not as a complement to food but rather as an alternative thereby shaping public perception. This study seeks to provide a multidisciplinary contribution to literature at the intersection of communication and nutrition/dietary sciences. Ensuring that social and digital communication channels operate parallel with evidence-based scientific and clinical studies can enhance the accuracy, reach, and effectiveness of public guidance. Such coordinated dissemination of knowledge plays a pivotal role in directing individuals toward safe, appropriate, and scientifically informed supplement practices.

## 1 Introduction

In recent years, functional foods have emerged as important approaches that complement modern medicine and support health (1). Functional foods are defined as foods that not only meet nutritional needs but also provide health-improving, protective, or disease-reducing effects. Terminologically, the concept of functional foods refers to foods that contain biologically active components beyond their nutritional value and offer specific health benefits (2). Phytochemicals, polyphenols, flavonoids, and various antioxidants are prominent among these components (3).

The functional foods market is experiencing significant growth driven by advances in food technology and increased consumer awareness of the relationship between nutrition and health. Encapsulation methods and innovative approaches to enhance bioavailability are enhancing the effectiveness of functional ingredients, making it possible to offer these products in a more accessible and effective form (64).

Advances in biotechnology and nutritional science have accelerated extensive research into the health effects of dietary supplements, strengthening the scientific evidence for antioxidants, phytochemicals, probiotics, and other bioactive compounds (4). These advances have fueled the growth of the global nutraceutical industry, which has strong markets in the US, Japan, and many European countries. The sector has begun to encompass a broad range of products, including dietary supplements, functional foods, and herbal products (64).

Socioeconomic developments, technological advancements, and lifestyle changes are leading to increased exposure to health risks, increasing the need for societies to strengthen community nutrition strategies. In this context, functional foods and nutritional supplements have become a central topic of current research and industrial development for both health protection and disease prevention (5).

In the post-pandemic period, the growing interest in functional foods and dietary supplements reflects not only individual pursuits for improved health but also broader shifts in global public-health practices. During the COVID-19 pandemic, the motivation to enhance immune function led to a marked increase in supplement consumption, with numerous studies reporting a sharp rise in usage rates across many countries compared with pre-pandemic levels (6). This trend has simultaneously stimulated a surge in digital content production related to dietary supplements, particularly on social media platforms, thereby situating the circulation of supplement-related information increasingly within online ecosystems (65). The health communication ecosystem that people experience daily has changed significantly and is now more complex and sophisticated than ever before. Broadly speaking, the health communication ecosystem encompasses the countless dynamic and diverse individuals, networks, organizations, and structures that influence how people understand and experience health and illness through the production and sharing of health-related information. Within this communication ecosystem, individuals are exposed to diverse information (7).

Communication is a fundamental process through which individuals and groups exchange information, emotions, and meanings, facilitating understanding and interaction. It involves the encoding of a message by a sender, its transmission through a channel, and its decoding by a receiver, incorporating both verbal and nonverbal elements such as gestures, facial expressions, and symbols (8–10). Communication is inherently a dynamic and continuous process that is context-sensitive, meaning that the interpretation of messages is influenced by social, cultural, and situational factors (11, 12). Its key features include bidirectionality, the capacity to create shared meaning, and the use of multiple channels to achieve specific objectives (13, 14). The primary purpose of communication is to share meaning and facilitate interaction, which encompasses the transmission of information, persuasion, the establishment of social bonds, and the cultural transfer of norms and values (10, 14). Therefore, communication serves as both a practical and a social tool, essential for coordinating behavior, fostering relationships, and maintaining cultural continuity.

The digitalization of communication has significantly reshaped internet-based news reporting, transforming how public institutions, state agencies, and professional organizations disseminate information online. Internet news reporting emphasizes speed, accessibility, and interactivity, enabling organizations to reach broader audiences while maintaining official narratives (15). The shift from traditional broadcast channels to web-based platforms has introduced both opportunities and challenges: while digital tools allow real-time updates and multimedia integration, they also demand rigorous verification practices to prevent misinformation and maintain public trust (16, 17). Consequently, internet news reporting exemplifies the intersection of communication, technology, and institutional strategy, illustrating how digitalization reshapes the production, distribution, and reception of news in the contemporary information environment.

In the contemporary media landscape, traditional news outlets are increasingly being replaced by web-based news platforms. Among these, the BBC stands out as one of the most prominent global news organizations, offering wide-reaching coverage and serving as a key reference point in shaping public perceptions and attitudes. As such, internet journalism exemplifies the intersection of communication, technology, and institutional strategy, demonstrating how digitalization is reshaping the production, distribution, and reception of news in the modern information environment.

Today, individuals rely extensively on web-based sources for their primary information-seeking processes, and the digital platforms of news organizations have become critical reference points in shaping health-related perceptions and attitudes. Consequently, the content disseminated by mainstream news websites not only serves an informational purpose but also fulfills a directive and framing function within the broader digital communication environment (18). Within this context, the present study aims to systematically examine dietary-supplement content published on the official website of the BBC, one of the world’s most widely accessed and influential news organizations (19, 20). For this purpose, the platform’s internal search engine was queried using the keywords *supplement, nutraceutical and protein powder* and from the resulting items, 45 news texts directly related to dietary supplements were selected through a simple sampling procedure. The analysis was conducted using the Wordsmith software, which enabled the construction of a Word List and the identification of the most frequently occurring and context-bearing lexical items within the corpus (21). By examining the contextual patterns associated with these lexical items, the study identifies the discursive frames through which the BBC represents dietary supplements. These frames are interpreted in light of the nutrition and dietary supplement literature and further discussed within the context of digital health communication to evaluate their potential influence on public perceptions. Overall, this study seeks to address a notable gap in literature by highlighting the role of mainstream digital media in the production of information and the shaping of public understanding regarding dietary supplements in online environments.

Despite the growing body of research on health communication and media coverage of nutrition-related topics, an important gap remains in the literature regarding how dietary supplements are framed in news media from both a communication and a nutrition science perspective. Previous studies have primarily focused either on the frequency and tone of media coverage or on regulatory and risk communication aspects of supplements. However, limited attention has been given to integrating media framing analysis with a nutrition-based evaluation of the products discussed in news content. In particular, how different types of dietary supplements are presented, which nutritional claims are emphasized, and whether these representations align with scientific evidence remain underexplored. Therefore, this study aims to address this gap by combining media framing analysis with a nutrition-oriented assessment of dietary supplements featured in news articles, thereby providing a more comprehensive understanding of how such products are communicated to the public.

From a nutrition science perspective, dietary supplements are generally defined as products intended to complement the diet rather than replace balanced and adequate nutrition (22). The fundamental principles of nutrition emphasize that nutrient requirements should primarily be met through a diverse and balanced dietary pattern (23). Although dietary supplements may be beneficial in specific cases such as nutrient deficiencies, certain physiological conditions, or medically indicated situations, excessive or unnecessary consumption may lead to potential health risks (24). In recent years, the growing market of dietary supplements and their frequent representation in media content have raised concerns about whether the information presented to the public reflects evidence-based nutritional guidance (25). Therefore, examining how supplements are communicated in the media is important not only from a communication perspective but also from a public health and nutrition standpoint. Understanding the ways in which supplements are framed in news content may provide insights into how media narratives influence public perceptions regarding nutritional adequacy, supplement necessity, and potential health risks.

## 2 Theoretical framework

### 2.1 Content and framing analysis

Content analysis is a versatile and robust research methodology that enables scholars to derive systematic insights into a particular issue or phenomenon and to formulate empirically grounded interpretations based on these insights. It has become a widely employed technique in the social sciences, largely because it accommodates both qualitative and quantitative examinations of mass media content. Conceptually, content analysis is defined as a systematic, methodological, and objective procedure for identifying, categorizing, and interpreting the essential components embedded in textual or discursive data. Its central purpose is to analyze the presence, distribution, and meaning of predefined categories within a given text (26).

Content analysis has often been described as the process through which written expressions are quantified and transformed into analyzable data. At its core lies the categorization of textual elements and the measurement of their frequency (27). This method offers researchers rich interpretative potential by enabling the systematic examination of written material (28), p. 133. In practice, conducting content analysis requires first identifying the corpus to be analyzed and subsequently developing a coding system that captures selected features of the content. The frequency of specific words or themes is then systematically recorded. Within this study, the news texts comprising the sample will be processed through Wordsmith software, which will generate word lists that reveal the most frequently occurring lexical items in the corpus. In this way, the dominant concepts structuring the news discourse will be identified. Accordingly, the study adopts a quantitative content analysis approach.

Quantitative content analysis primarily aims to determine the frequency of occurrence of words or concepts that have been operationalized during the category development phase. Because this technique predominantly concentrates on coding the manifest surface content of texts, it typically employs open coding procedures. For instance, the number of times a specific word or concept appears in a text is recorded using coding schemes that treat the presence or absence of the target item as the definitional criterion. The reliability of this approach is generally high, and computerized tools are frequently utilized to enhance accuracy and efficiency (29), p. 470. In this study, the Wordsmith software served as the primary analytical tool.

Content analysis aims to provide an objective, measurable, and verifiable account of the explicit content of messages (30), p. 176. Adhering to a rigorous and consistent methodological structure is therefore essential. Establishing clear analytical parameters, categorizing the data accordingly, and ensuring replicable results constitute critical components of effective content analysis. Moreover, situating textual elements within a specific semantic and interpretive framework is fundamental to achieving analytical depth (31), pp. 21–23. For this reason, the present study also employs the concordance (*context*) feature of the Wordsmith software to examine the contextual environments in which frequently occurring lexical items appear. These contextual analyses will elucidate the discursive patterns and thematic orientations embedded in the news texts. For this reason, another method used in this study, apart from content analysis, was framing analysis.

Framing analysis is conceptualized as a constructivist methodological paradigm for interrogating news discourse, wherein news texts are systematically deconstructed into empirically operationalizable dimensions—encompassing syntactical, script, thematic, and rhetorical structures—thereby facilitating the rigorous identification and substantiation of the mechanisms through which the news media actively construct and delineate interpretive frames around salient issues (32). Within the domain of mass communication research, framing analysis occupies an epistemological position intermediary to discourse analysis and content analysis, typically integrating both qualitative and quantitative.

## 3 Materials and methods

### 3.1 Sample selection

The BBC is one of the world’s leading public-service broadcasters. Headquartered in the United Kingdom, the BBC produces original, world-class programs and content that inform, educate, and entertain millions of people worldwide (33). Through the BBC World Service, it broadcasts on television, radio, and online in over 40 languages, reaching a diverse international audience (33). BBC News, primarily via the World Service, reaches approximately 418 million viewers weekly, highlighting its extensive global reach (34). Currently, the BBC World Service operates in 43 languages, including English, and maintains correspondents and support staff in 64 cities across 54 countries, providing audiences with the global insight and expertise necessary to deliver a truly international news service (35). This extensive infrastructure positions the BBC as a highly influential global news platform, making it a critical reference point in the dissemination and framing of information in the contemporary digital media landscape. Therefore, the BBC was chosen as the sample for this study. The sample was identified using keywords from www.bbc.com. In this context, the *search* section of www.bbc.com searched for *supplement*, *nutraceutical* and *protein powder*. This search yielded 416 news items. The number of words in these news texts is 39,740. From these news items, 45 news items were selected using a simple sample selection method, taking into account the emphasis in the news headlines, and constituted the sample for this study.

The search terms used in this study (*“dietary supplement,” “nutraceutical,”* and *“protein powder”*) were determined on the basis of the most commonly employed concepts in both the academic literature and media discourse. The terms *“dietary supplement”* and *“food supplement”* represent the primary concepts used in regulatory and legal frameworks. The concept of *“nutraceutical,”* by contrast, is a technical term frequently used in the scientific literature to describe products with functional and therapeutic purposes. The term *“protein powder,”* meanwhile, refers to a specific yet representative product category that has high visibility in the media, particularly in the context of sports nutrition and performance-oriented consumption. The combined use of these terms aims to facilitate a more comprehensive analysis of media representations by encompassing both the general category level and the level of specific products. The selection of these terms was structured by the researchers on the basis of a review of the existing literature and the frequency of conceptual usage within the relevant field.

In this study, the temporal scope of the analyzed news items was not determined through the prior specification of a fixed time period; rather, it emerged as a natural outcome of the data collection process. Within the scope of the research, the search function available on the official website of BBC was used to conduct separate searches using the keywords *“supplement,” “nutraceutical,”* and *“protein powder.”* News items retrieved through these keyword searches were screened, and those relevant to the aims and scope of the study were included in the sample. The search terms were entered individually into the search engine of the BBC’s website: www.bbc.com. Each term was queried independently, and the resulting outputs were subsequently merged, after which duplicate items were removed.

Only news articles published in English were included in the study. This preference is based on the assumption that the BBC’s global English-language publications represent an international media framework. No additional restrictions were imposed with regard to author type, article length, news category, or publication format. Accordingly, all types of news content (e.g., analyses, interviews, health reports) were included in the dataset. However, duplicate items and texts that did not conform to the news article format (such as video transcripts or gallery descriptions) were excluded from the analysis.

As a result of this procedure, the publication dates of the selected news items were found to range from 14 October 2014 to 5 November 2025. Therefore, the temporal limitation of the study was not established through the prior determination of a specific time frame; rather, it was defined by the period during which news content meeting the research criteria and retrievable through the specified keywords was published. Accordingly, the dataset of the study is limited to news articles published within this period and accessible through the aforementioned keywords.

### 3.2 Methods

The methodological design of this study was operationalized through a five-stage analytical protocol. First, an initial corpus of 416 news items was retrieved via a keyword-based search on the BBC website. From this corpus, a probabilistic sample of 45 news items was drawn using a simple random sampling procedure, constituting the study’s unit of analysis.

Second, all textual data underwent a standardized pre-processing phase to ensure analytical reliability. The corpus was digitized and converted into Wordsmith-compatible plain-text (.txt) files. During this stage, non-linguistic elements—including typographical redundancies, headings, pagination markers, and extraneous metadata—were systematically removed to minimize noise and prevent coding distortion.

Third, lexical profiling of the corpus was conducted using the WordList module of WordSmith Tools. Token and type counts were generated for each text to establish baseline lexical density and distribution patterns. Lexical items with a frequency threshold exceeding 10 occurrences were retained for further analysis. Conversely, function words (e.g., conjunctions, adverbs, morphological suffixes) that lacked contextual or semantic load were excluded to refine the analytical focus.

In the fourth stage, the retained lexical items were subjected to concordance analysis using the Concord module. Through examination of right- and left-hand co-text environments, recurrent thematic and discursive structures were identified. This step enabled the systematic detection of framing patterns embedded in the news discourse.

Finally, the concordance outputs were interpreted within the study’s analytical framework, focusing on: (1) Which types of food supplements are most frequently featured in news content? (2) Is there a scientific basis? (3) Is the benefit or the risk more prominent? (4) How does social media shape nutritional behavior? (5) Which target group does the news report address?

This study not only analyzes media content at a descriptive level but also examines it based on framing theory. Framing theory posits that the media structures public perceptions, risk assessments, and attitudes by presenting events and issues within specific meaning patterns (36, 66). According to Entman (36), framing is a selective emphasis process that includes (i) defining a problem, (ii) providing a causal interpretation, (iii) making a moral assessment, and (iv) proposing a solution. In this context, the media is not merely a neutral tool that transmits information; it is an actor that produces and directs meaning.

In the health communication literature, framing is widely used, particularly in the context of risk–benefit presentation, scientific ambiguity, attribution to expert authority, and audience construction (67, 68). In hybrid product categories related to health, consumption, and performance, such as dietary supplements, media framing can directly influence consumer perception and behavior. This study analyzes BBC news through the following framing dimensions (Table 1).

**Table 1:** Framing themes and analytical questions used in the analysis of food supplement news content.

Framing theme	Analysis question
Food supplement category frame	Which types of food supplements are most frequently featured in news content?
Scientific frame	Is there a scientific basis?
Benefit–risk balance frame	Is the benefit or the risk more prominent?
Social media frame	How does social media shape nutritional behavior?
Target audience frame	Which target group does the news report address?

These dimensions are structured in accordance with a mixed analytical approach that uses both content analysis and framing analysis together.

### 3.3 Theoretical justification of research questions

1 Which types of food supplements are most frequently featured in news content?

Media visibility is one of the key elements that determine the societal importance attributed to a topic (37) (Agenda Setting Theory). More frequent appearance of certain product categories can lead to those products becoming more prominent in the public eye in terms of risk or benefit. Therefore, the distribution of product types is important in showing which areas the media prioritizes.

2 What is the scientific basis of the information presented in news content?

The representation of scientific evidence in health news is a critical factor in terms of public trust and health literacy (38). The lack or oversimplification of scientific references in the media can create a false perception of risk. In the context of framing theory, scientific citation serves as a function of “causal interpretation” and “legitimization.” Science Communication.

3 Are benefits or risks emphasized more?

In health communication, the emphasis on gain and loss frames affects individuals’ behavioral intentions in different ways (39, 40). In semi-medicinal products such as food supplements, the risk–benefit frame can directly shape consumption behavior. Therefore, which aspect is emphasized in the news is a fundamental dimension of analysis.

4 How does social media shape nutritional behavior?

The production of health information in the digital media environment is not limited to traditional media. Influencers and social media content play a decisive role in the formation of nutritional preferences, especially in young adults (41). The inclusion of social media references in traditional media content points to a hybrid framing structure.

5 Which target groups are addressed in the news?

In framing theory, audience construction shows who the message is intended for and who is positioned at risk (36). Highlighting specific groups such as athletes, the elderly, children, or women reveals how societal risk distribution is represented. Within this theoretical framework, this study aims to systematically analyze how food supplements are structured in BBC news, the semantic patterns within which they are presented, and the kind of health discourse that is generated in the public sphere.

## 4 Result and discussion

Table 2 presents the word list derived from news articles on food supplements published on the BBC’s official website. Only words with a frequency of 10 or more were included, while conjunctions, adverbs, adjectives, and suffixes were excluded from the analysis. The data reveal several notable patterns.

**Table 2:** BBC news’ simplified word list.

N	Word	Freq.
1	PROTEIN	203
2	SAYS	181
3	PEOPLE	170
4	MORE	168
5	FOOD	144
6	SUPPLEMENTS	121
7	COLLAGEN	113
8	SLEEP	112
9	VITAMIN	111
10	HEALTH	109
11	SAID	105
12	CAFFEINE	91
13	D	90
14	TAKING	79
15	DIET	78
16	INSECTS	76
17	FOUND	75
18	RESEARCH	75
19	HIGH	66
20	FOODS	64
21	PRODUCTS	63
22	HELP	58
23	EVIDENCE	57
24	STUDIES	57
25	SKIN	53
26	MAGNESIUM	51
27	SUPPLEMENT	50
28	HEALTHY	49
29	POWDER	49
30	TIME	49
31	LEVELS	47
32	WEIGHT	45
33	SAY	43
34	INSECT	41
35	RISK	41
36	UNIVERSITY	40
37	IMPROVE	39
38	BRAIN	38
39	AMOUNT	33
40	DAILY	32
41	RECOMMENDED	32
42	TRIALS	32
43	DR	31
44	EFFECTS	31
45	ENOUGH	31
46	IMPORTANT	31
47	INGREDIENTS	31
48	RESEARCHERS	31
49	SPIRULINA	31
50	STUDY	31
51	PRODUCT	30
52	BENEFIT	28
53	PLANT	28
54	ENERGY	27
55	RICH	27
56	VITAMINS	27
57	ANIMAL	26
58	CLAIMS	26
59	NUTRITION	26
60	PROTEINS	24
61	SCIENTISTS	24
62	EFFECT	23
63	GREEN	23
64	GUT	23
65	MUSCLE	23
66	PROF	23
67	TAURINE	23
68	INCREASE	22
69	POWDERS	22
70	YOUNG	22
71	HUMAN	21
72	MOLECULE	21
73	PILLS	21
74	PRODUCTION	21
75	TRIAL	21
76	CLINICAL	20
77	COFFEE	20
78	CONTAIN	20
79	DIETS	20
80	DISEASE	20
81	GIVEN	20
82	ACIDS	19
83	ACTUALLY	19
84	BABIES	19
85	CREATINE	19
86	ESPECIALLY	19
87	MELATONIN	19
88	NUTRIENTS	19
89	REDUCE	19
90	TRYING	19
91	ADVICE	18
92	BODIES	18
93	CANCER	18
94	CAUSE	18
95	COMPANIES	18
96	CONSUMING	18
97	FORM	18
98	GIVE	18
99	GYM	18
100	IMPACT	18
101	LOT	18
102	MINERALS	18
103	PROCESS	18
104	PUT	18
105	START	18
106	VEGETABLES	18
107	ACCORDING	17
108	AGAIN	17
109	AMINO	17
110	CONSUMPTION	17
111	DIFFERENCE	17
112	EDIBLE	17
113	HEART	17
114	HELPS	17
115	HOSPITAL	17
116	KEEP	17
117	KNOWN	17
118	LOW	17
119	MAKING	17
120	POTENTIAL	17
121	BONES	16
122	CHILDREN	16
123	COMPARED	16
124	CONSUME	16
125	CONSUMERS	16
126	FOCUS	16
127	INDUSTRY	16
128	MEALS	16
129	RANGE	16
130	SHORT	16
131	STOP	16
132	SUGGESTS	16
133	ADDED	15
134	ESSENTIAL	15
135	FIGHT	15
136	HARD	15
137	LEAD	15
138	MEAT	15
139	NIGHT	15
140	NUTS	15
141	PROFESSOR	15
142	WHEY	15
143	CONDITIONS	14
144	CONTAINING	14
145	DIETARY	14
146	LOWER	14
147	MUSCLES	14
148	NUTRIENT	14
149	NUTRITIONAL	14
150	OVERALL	14
151	POTENTIALLY	14
152	PROCESSED	14
153	SEEMS	14
154	SEVERAL	14
155	SOCIAL	14
156	SPECIES	14
157	SYMPTOMS	14
158	WOMEN	14
159	ADULTS	13
160	DEATH	13
161	DIFFICULT	13
162	DOSE	13
163	DRUGS	13
164	LEAST	13
165	MEDIA	13
166	MOLECULES	13
167	NATURALLY	13
168	NEEDED	13
169	RESULTS	13
170	RISKS	13
171	SAFETY	13
172	TIMES	13
173	VIVID	13
174	WORKING	13
175	BIG	12
176	BONE	12
177	CALORIES	12
178	CARE	12
179	CASE	12
180	CONFERENCE	12
181	CONSTIPATION	12
182	DOSES	12
183	DRIED	12
184	FACT	12
185	FUTURE	12
186	GROW	12
187	GROWING	12
188	INCREASED	12
189	INSTEAD	12
190	INVOLVED	12
191	KEY	12
192	LOOKING	12
193	MAKES	12
194	PILL	12
195	POSSIBLE	12
196	PRE	12
197	PRODUCED	12
198	REDUCING	12
199	ROOM	12
200	SAFE	12
201	SHOWED	12
202	SUGGEST	12
203	AMERICAN	11
204	ASSOCIATED	11
205	AVAILABLE	11
206	BEGAN	11
207	BOOST	11
208	CAN’T	11
209	CHANGE	11
210	CONTAINS	11
211	DAIRY	11
212	GUIDANCE	11
213	JOINTS	11
214	LINKED	11
215	LIVESTOCK	11
216	LOSE	11
217	MEANS	11
218	MEANWHILE	11
219	MEDICAL	11
220	NATURAL	11
221	REPLACEMENT	11
222	REPORT	11
223	SCIENCE	11
224	SCIENTIFIC	11
225	STANDARDS	11
226	TRAINING	11
227	TYPES	11
228	ACROSS	10
229	ADENOSINE	10
230	ADVISED	10
231	AMOUNTS	10
232	ANIMALS	10
233	ANTIOXIDANTS	10
234	APPETITE	10
235	ATHLETES	10
236	AVERAGE	10
237	BATCHES	10
238	BENEFICIAL	10
239	COVID	10
240	DARK	10
241	GLOBAL	10
242	IMPROVED	10
243	PATIENTS	10
244	POOR	10
245	PROBABLY	10
246	PUBLIC	10
247	STRESS	10
248	STRUCTURE	10
249	SUSTAINABLE	10
250	TEST	10

According to Table 2, the most frequently occurring word in the corpus is *protein*, a term classified as a type of food supplement, whose prominence may be interpreted as an indicator of the corpus’s integrated thematic focus. The word *says* appears with a frequency of 181, making it the second most frequent lexical item, suggesting a strong reliance on expert statements within the news texts. The additional frequency of related forms such as *said* (105) and *say* (43) further supports this interpretation, as does the presence of terms including *scientists*, *clinical*, *professor*, *evidence*, *university*, *studies*, *improve*, *claim*, *health*, *public*, *research*, *report*, and *test* in the word list. The frequent appearance of items such as *food* (144), *supplements* (121), *collagen* (113), *vitamin* (111), *caffeine* (91), *D* (90), *magnesium* (51), *powder* (49), *spirulina* (31), *vitamins* (27), *proteins* (24), *taurine* (23), *melatonin* (19), *creatine* (19), *minerals* (18), and *whey* (15) provides further insight into the diverse categories of food supplements represented in the corpus. The texts also include phrases indicating the target audience, reflected in the notable frequency of words such as *adult*, *adults*, *children*, *women*, *young*, and *babies*. Additionally, the distribution contains terms with both positive and negative connotations—such as *risk*, *benefit*, *effect*, *death*, *dose*, and *safety*—which constitutes another noteworthy dimension of the dataset.

In this study, Wordsmith content and framing analysis was applied to selected news articles from the BBC, and the findings were systematically evaluated according to five pre-determined thematic frameworks (Table 3). Discursive patterns were examined during the analysis process, specifically to understand the relationships between nutrition, supplement use, and media discourse. Within this scope, news articles were interpreted along the following axes:

**Table 3:** Thematic frameworks used in Wordsmith content and framing analysis.

No	Framing theme	Analysis question	Words	Duality
1	Food supplement category frame	Which types of food supplements are most frequently featured in news content?	Supplements, Nutrition, Nutrients, Vitamins, Minerals, Amino acid, Magnesium, Collagen, Protein, Powder, Caffeine, Taurine Plant, Spirulina, Melatonin, Creatine, Antioxidant, Whey	Strong emphasis
2	Scientific frame	Is there a scientific basis?	Scientists, Clinical, Professor, Evidence, University, Studies, Improve, Claim, Health, Public, Research, Report, Test	Strong emphasis
3	Benefit–risk balance frame	Is the benefit or the risk more prominent?	Risk, Benefit, Effect, Death, Dose, Safety	Strong emphasis
4	Social media frame	How does social media shape nutritional behavior?	Social, Media	Weak emphasis
5	Target audience frame	Which target group does the news report address?	Adult, Adults, Children, Women, Young, Babies	Strong emphasis

The findings were presented in line with the guiding structure of these thematic questions. Thus, the discursive frameworks used to structure news content and how these frameworks contribute to the production of meaning regarding community health care nutritional perception, and supplement use were holistically revealed.

**Figure 1:**
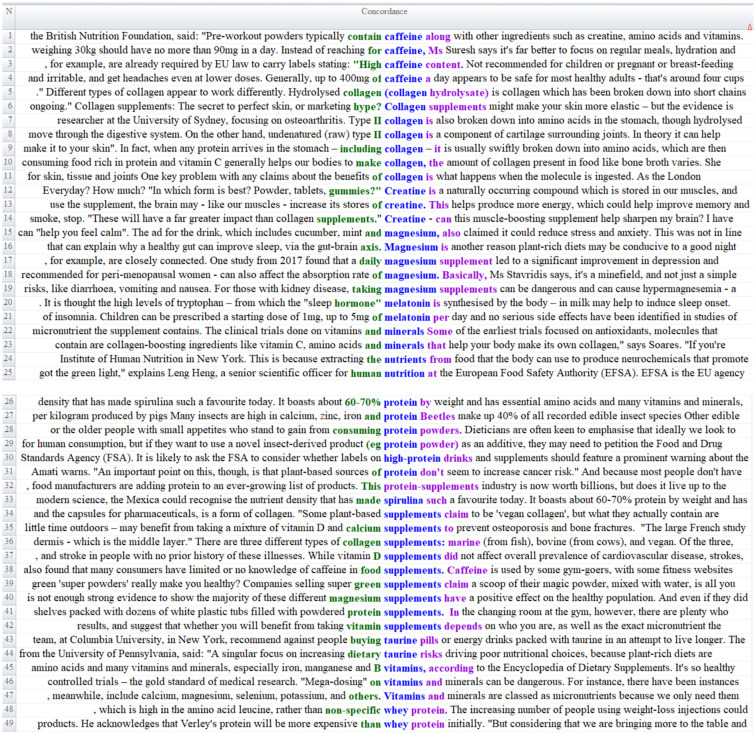
Content analysis of food supplement category frame.

As a result of the content analysis of the news texts, it was determined that the keywords supplements, nutrition, nutrients, vitamins, minerals, amino acid, magnesium, collagen, protein, powder, caffeine, taurine, spirulina, melatonin, creatine, antioxidant and whey within the scope of the *Food Supplement Category Frame* theme were the most frequently used concepts and the context sentences are given in Figure 1.

According to Figure 1, the BBC news reports indicate that supplements used by athletes contain creatine, amino acids, vitamins, and caffeine (Figure 1, Line 1). The reports further underscore the importance of consuming regular meals and adequate water instead of foods containing caffeine (Line 2), thereby emphasizing healthy dietary practices. The news texts additionally highlight various regulations concerning supplements and reference relevant European Union legislation (Lines 3, 25). Expert opinions on caffeine consumption are also presented, accompanied by recommendations regarding optimal daily caffeine intake (Lines 4, 38).

Collagen emerges as another prominent supplement within the news coverage. The reports provide technical information about collagen (Lines 5, 8, 36), outline its purported benefits (Lines 6, 9), and identify food products that may naturally meet the body’s collagen requirements (Line 10). In addition to collagen, the texts also draw attention to the use of creatine (Lines 13, 14), magnesium (Lines 16–19), melatonin (Lines 20–21), as well as calcium, selenium, potassium (Line 47), vitamin D (Lines 35, 37), and whey protein (Lines 48–49). The active ingredients of food supplements are similarly discussed, including technical information on Spirulina’s protein content, essential amino acids, vitamins, and minerals (Lines 26, 33). The news reports also address popular topics such as edible insects and plant-based proteins (Lines 27, 29, 31, 34). Some criticisms concerning the increasing trend in supplement consumption are likewise included (Line 41). In this context, it is noted that the positive or negative health effects of supplementary foods remain scientifically inconclusive (Lines 39, 40).

This framing of the food supplements category demonstrates that the content in news articles largely aligns with existing scientific literature. Protein powders, collagen, magnesium, melatonin, caffeine, creatine, taurine and vitamin D and mineral supplements are among the most frequently discussed product groups in both popular culture and academic research (42). Dose restrictions outlined in news reports about caffeine consumption align with the safe intake levels established by the European Food Safety Authority (EFSA) (43) and FDA, with lower tolerance levels recommended for children, adolescents, and pregnant women (44). Similarly, the efficacy and safety profiles of melatonin, magnesium, and collagen supplements reflect the differences in evidence levels highlighted in the literature (45). For example, academic sources have cited studies on the differential effects of different types of collagen peptides on skin elasticity, muscle, bone, and joint health (46). The neurocognitive effects presented in news reports about creatine supplements are consistent with recent clinical studies demonstrating the beneficial effects of creatine on brain energy metabolism beyond muscle function (47). Furthermore, the potential ameliorative effects of magnesium on sleep quality, stress, and depression symptoms have been supported by a growing number of randomized controlled trials in recent years (48). However, as highlighted in the news, misuse, overdose, the risk of toxicity in individuals with renal impairment, and the lack of scientific basis for product marketing claims are considered critical community nutrition and health issues by both international regulatory authorities and researchers (69). Therefore, academic literature generally recommends the use of dietary supplements in controlled doses, with evidence-based rationale, and under professional guidance (49).

**Figure 2:**
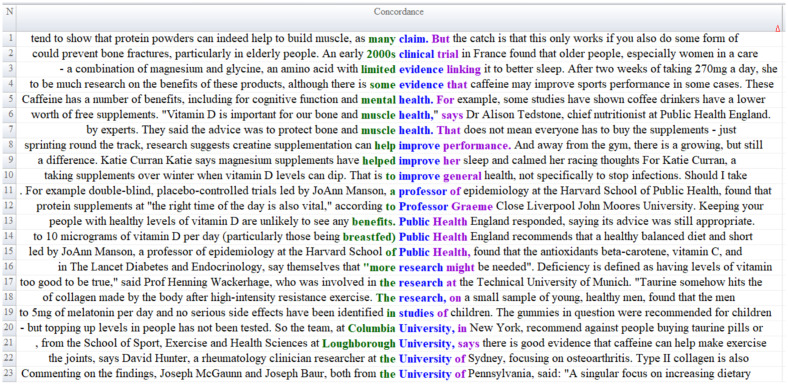
Content analysis of scientific frame.

Figure 2 presents the contextual distribution of the words constituting the *Scientific Frame*. According to the data in Figure 2, BBC news reports suggest that the use of protein and creatine contributes to muscle development and enhances physical performance (Figure 2, Lines 1 and 8); that dietary supplements may help prevent bone fractures in older adults (Line 2); that magnesium and glycine promote improved and healthier sleep (Line 3); and that caffeine intake can enhance athletic performance while also offering cognitive and mental health benefits (Lines 4 and 5). The reports further underscore the positive evaluations provided by experts from official bodies such as community ealth England regarding the use of supplements (Lines 6, 7, 9, 11, 12, 17, 21, 22, and 23). Additionally, the news texts emphasize that dietary supplements support overall health (Line 10) and include expert commentary on the appropriate use of such supplements (Lines 12 and 14).

An analysis of the findings reported in news articles and expert opinions reveals that the evidence regarding the effectiveness and safety of dietary supplements is heterogeneous. While relatively robust clinical and sports science data exist for performance-enhancing supplements such as protein powders, creatine, and caffeine, the level of evidence for other supplements, such as collagen, melatonin, spirulina, and various herbal products, is understood to be limited (50).

Furthermore, findings for essential micronutrients such as vitamin D and magnesium vary depending on age group, health status, and lifestyle, emphasizing that the potential benefits are particularly pronounced in older adults and individuals at risk of deficiency (51). However, the frequent misuse, overdose, inappropriate consumption in children, and unfounded claims frequently cited in news reports highlight the importance of personal responsibility and expert guidance in supplement use (52). Consistent with academic literature, dietary supplements should only be used based on individual needs, evidence base, and scientific guidelines, while a healthy diet should be maintained as the primary strategy.

**Figure 3:**
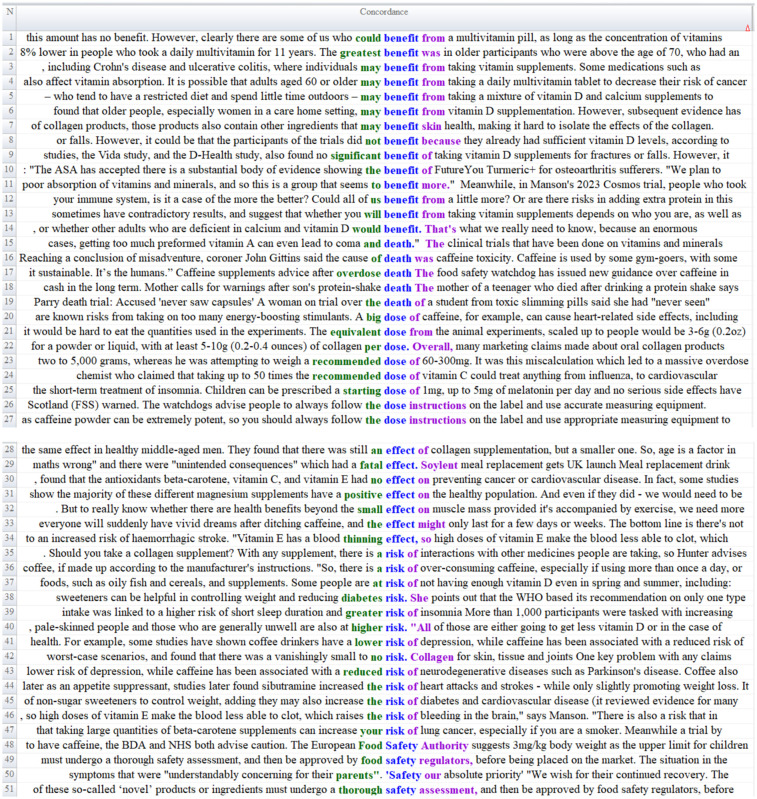
Content analysis of benefit–risk balance frame.

Considering the contextual results presented in Figure 3, it can be argued that one of the prominent frames in the news texts is constructed around the balance between benefits and risks. The contexts of the word *benefit, death, dose, effect, risk, safety—*which appears with high frequency in the word list—underscore several themes: that multivitamin use provides general health benefits (Figure 3, Lines 1–2); that vitamin supplements, drawing on expert knowledge, are beneficial for a range of diseases (e.g., ulcerative colitis, cancer; Lines 3–4); that vitamin D and calcium supplements support dietary intake (Line 5); that vitamin D use is advantageous for specific target groups (e.g., women over 60; Lines 6, 8–9); and that collagen supplementation contributes to skin health (Line 7).

In addition to the benefit-oriented framing of food supplement use, the news texts also highlight potential harms. In this regard, several reports indicate that the use of certain supplements can result in fatal outcomes (Line 15). The texts further emphasize the potentially lethal consequences of excessive caffeine consumption, protein shakes, and meal replacement products (e.g., Soylent; Lines 16–18, 29), as well as the adverse side effects of overstimulating energy-enhancing products that may trigger heart disease (Line 20). The potential risks of supplement use are likewise foregrounded in relation to dietary practices (Lines 44–45). Moreover, another notable emphasis within the benefit–risk framework concerns the appropriate dosage of dietary supplements. The articles frequently offer dosage recommendations based on scientific evidence (Lines 21–26).

The benefit–risk framework also includes contradictory statements. While some researchers argue that dietary supplements are beneficial for certain diseases (Lines 3–4), other users contend that supplements have no effect on preventing cancer or cardiovascular disease (Line 30). Similarly, several articles assert that the purported health benefits of dietary supplements have not been conclusively demonstrated (Line 32). Some reports additionally include explicit health claims, such as *Vitamin E use reduces blood clotting* (Lines 34, 46). Supplement use is also discussed in connection with various disciplines; for example, the texts reference studies highlighting the relationship between coffee consumption and depression (Line 41).

Another noteworthy aspect within the benefit–risk framework is the reference to *food safety regulators* (Line 51). The emphasis on *safety* in supplement consumption is prominent throughout the articles (Lines 49–50), which also cite official authorities responsible for ensuring food safety (Line 48).

This coexistence of supportive and skeptical narratives reflects a contradictory framing structure frequently observed in media coverage of nutrition-related topics. Similar patterns have been reported in previous studies examining media representations of dietary supplements and functional foods, where benefits are often presented alongside cautionary statements about safety and effectiveness (53).

Official regulatory bodies also focus attention on dosage and safety: EFSA (European Food Safety Authority) emphasizes the importance of keeping bioactive substance concentrations within safe limits for many supplement ingredients and highlights the potential harms of excessive consumption (43).

Daily doses of food supplements and dietary supplements are calculated within specific minimum and maximum limits to protect individual health and prevent potential side effects (54). These values are generally based on scientific principles such as Dietary Reference Values (DRVs), Population Reference Intakes (PRIs), Average Requirements (ARs), Adequate Intakes (AIs), and Reference Intake Ranges (RIs). Minimum values represent the level that will meet an individual’s essential nutrient requirements, while maximum values are determined to prevent toxicity and side effects that may occur from excessive consumption (43). International and national health authorities and regulatory bodies are responsible for setting these dose limits. In Europe, this task is primarily carried out by the European Food Safety Authority (EFSA), which determines safe upper limits (UL) and recommended daily intakes based on the panel’s scientific assessments. Globally, the World Health Organization (WHO) and the Food and Agriculture Organization of the United Nations (FAO) provide international recommendations and guidelines for macronutrients and micronutrients. Similarly, in the US, the Recommended Dietary Allowances (RDA) and Tolerable Upper Intake Levels (UL) established by the Institute of Medicine (IOM) are considered reference values for dietary supplements. In this framework, establishing minimum and maximum dietary supplement doses is both based on scientific evidence and ensures safe and effective supplement use (43).

Dietary reference values (DRVs) are a general term encompassing population-level recommended intakes for macronutrients and micronutrients and determine the amounts of nutrients that healthy individuals should regularly consume. In 2005, the European Commission commissioned EFSA to review SCF’s 1993 DRV recommendations for the European population with up-to-date scientific evidence. This task was entrusted to the EFSA Panel on Dietetic Products, Nutrition and Allergies (NDA), which published opinions in 2010 that addressed the general principles for the derivation and application of DRVs. Over the following 7 years, a total of 32 scientific opinions were issued, covering water, fats, carbohydrates, dietary fiber, protein, energy, and 14 vitamins and 13 minerals. The summary report brings together the results of these opinions with tables and appendices, making the DRVs readily available to end users (43).

The presence of regulatory references in news texts suggests that journalists attempt to enhance the credibility of health-related information by referring to scientific authorities such as the European Food Safety Authority. However, the findings of this study indicate that these references are often used selectively and superficially. Rather than explaining the scientific basis of regulatory frameworks, recommended intake levels, or the rationale behind safety limits, news articles tend to mention regulatory bodies mainly in the context of general safety warnings.

From a public health communication perspective, integrating academic evidence and official regulatory guidance more comprehensively into news reporting would be highly beneficial. Providing clearer explanations of regulatory recommendations and evidence-based intake limits could improve public awareness and encourage responsible supplement use. In this sense, referencing reliable scientific and institutional sources in a more informative manner may not only strengthen the credibility of nutrition-related news but also help guide individuals toward more informed health decisions.

**Figure 4:**
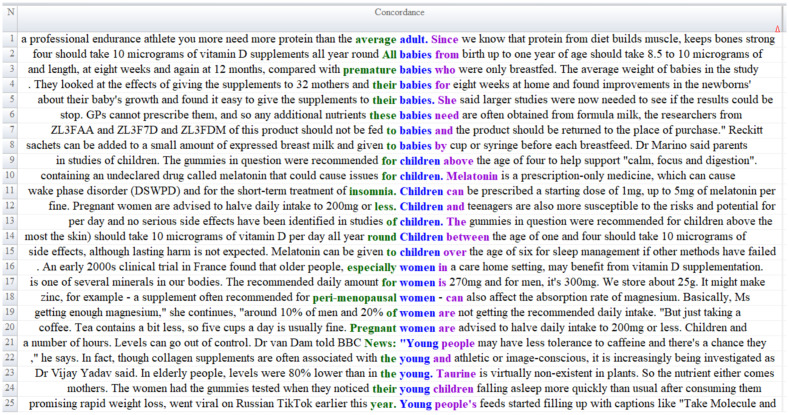
Content analysis of target audience frame.

The contextual data presented in Figure 4 indicates that specific target groups are referenced within the news coverage on food supplements. The news texts report that individuals who engage in regular exercise require higher protein intake than the general population and that protein consumption contributes to muscle and bone strengthening (Figure 4, Line 1). They further note that infants should receive 8.5–10 micrograms of vitamin D daily from birth until 1 year of age (Line 2), and that scientific studies have examined the use of food supplements among mothers and newborns (Lines 4–5). Additional statements highlight that infants require supplementation (Line 6) and that certain scientifically based studies suggest that some supplements may promote *calmness, focus, and digestion* in children (Line 9). The texts also warn against supplements containing ingredients harmful to children, such as melatonin (Line 10), although they note that such substances may be prescribed in controlled doses under specific conditions (Line 11). Moreover, the findings emphasize encouragement toward vitamin D consumption (Line 14); acknowledge that some supplements, despite potential side effects, do not cause permanent harm and may therefore be appropriate for particular target groups (Line 15); and report that recommendations regarding food supplements are supported by scientific evidence (Line 16). Finally, the data indicates that certain supplements, such as collagen, are associated with specific target populations, particularly young and athletic individuals (Line 22).

The contextual data presented in news articles highlights the importance of evaluating the use of dietary supplements across different target groups from a scientific nutrition perspective. Considering target age groups when using dietary supplements is critical from a nutritional and community nutrition perspective (55). Metabolic requirements of different age groups vary depending on their growth and developmental processes, immune system function, and lifestyle (56). This situation demonstrates the importance of planning supplementation strategies appropriate for target age groups in terms of both effectiveness and safety (57).

The World Health Organization (WHO) emphasizes the critical importance of planning food supplements according to target age groups. This strategic approach not only increases effectiveness but also plays a significant role in safety (58). WHO guidelines recommend analyzing the nutritional status and prevalence of micronutrient deficiencies in relevant population groups before implementing supplementation programs, carefully determining the doses of micronutrients used, and regularly monitoring potential side effects throughout the program. This approach provides a scientifically based framework for meeting age-specific nutritional needs and minimizing risks (59).

UNICEF’s report “Food-Based Dietary Guidelines: A Review of National Guidance for Children, Adolescents, and Women” (2021) provides important conclusions highlighting that dietary supplements require different strategies depending on their target group (*children, adolescents, pregnant and lactating women*) (60).

**Figure 5:**
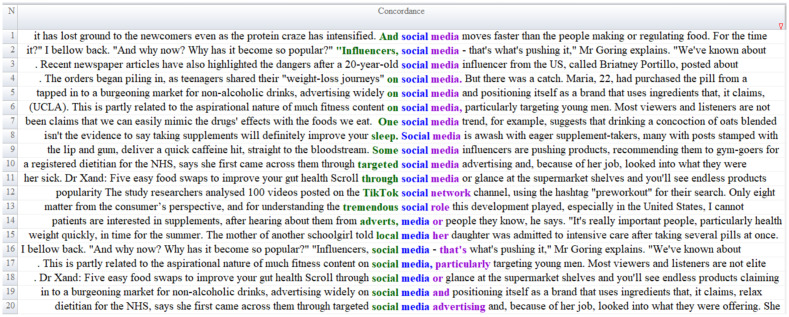
Content analysis of social media frame.

An examination of the context table for the terms *social* and *media* indicates that the use of protein supplements is increasingly promoted through social media platforms (Figure 5, line 1). Influencers play a prominent role in amplifying the popularity of food supplements (line 2), frequently producing advertisements that emphasize these products (line 9). Social media content related to *weight loss* is also prevalent (line 4), and fitness-oriented posts concerning supplement use circulate widely (line 6). Moreover, supplement-related posts often trend on these platforms (line 7), reflecting a substantial consumer base eager to purchase such products via social media channels (line 8). Advertising for supplements is disseminated extensively across social media (line 12), including platform-specific promotions on TikTok (lines 13 and 15). Finally, the potential risks associated with supplement consumption are communicated to the public through various media outlets (line 16).

The role of social media in dietary supplement and dietary supplement consumption is strongly confirmed in various academic studies. For example, a study of young adults in Kazakhstan found that social media was a key source of information on dietary supplement use (especially for muscle building and weight loss) among individuals aged 18–25. According to the study, 51% of participants cited social media as a key source of information, with Instagram and WhatsApp being the most frequently used platforms. This finding provides direct evidence that social platforms can drive demand for supplements through influencers and digital content (61).

Another academic study examines how digital media and social networks influence food supplement consumption among young adults (18–35 years) in Uttarakhand, India. Using a mixed-methods approach with 400 participants, the research finds that higher social media usage is positively associated with supplement intake, with Instagram and YouTube being the most influential platforms. User-generated content has a stronger effect on purchase decisions than branded content, and peer recommendations are more influential than celebrity endorsements (62). The study also identifies significant gender differences in platform influence. These results highlight the important role of digital media in shaping health product consumption in emerging markets, providing insights for marketers, policymakers, and community health strategies (63).

The findings of this study indicate that dietary supplements are frequently presented in news media through simplified and benefit-oriented narratives. While several supplements such as protein, creatine, caffeine, magnesium, melatonin, and collagen are discussed in relation to their potential physiological effects, the media coverage tends to prioritize practical benefits such as improved performance, energy, sleep quality, or esthetic outcomes. Although these representations are partly consistent with the scientific literature, the news framing often simplifies complex scientific evidence and rarely addresses the conditional nature of these benefits, including variability in individual responses, dosage considerations, or population-specific recommendations. As a result, the scientific nuances that typically accompany evidence-based nutritional guidance are largely absent from media narratives.

Another important limitation observed in the news coverage is the selective use of scientific and regulatory references. While some articles refer to expert opinions or regulatory authorities to legitimize claims, these references are often used in a fragmented manner and do not provide a comprehensive explanation of the safety frameworks governing dietary supplement use. In particular, issues such as appropriate dosage ranges, potential interactions with medications, and the risks associated with long-term or unsupervised consumption receive relatively limited attention. This selective presentation may contribute to an incomplete understanding of the role of dietary supplements as complementary components of nutrition rather than substitutes for balanced dietary patterns.

Furthermore, the dominance of benefit-oriented framing may have implications for public perceptions and consumption behavior. When supplements are repeatedly associated with desirable outcomes such as enhanced performance, improved appearance, or rapid health improvements, media narratives may unintentionally reinforce the perception that supplements represent accessible and efficient solutions to complex health concerns. Such representations risk obscuring the broader principles of nutritional adequacy and balanced dietary practices that constitute the foundation of public nutrition recommendations.

Taken together, these findings suggest that while news media play an important role in disseminating nutrition-related information, the framing strategies observed in the present study may contribute to the simplification of scientific evidence and the amplification of benefit-focused narratives. From a public health perspective, this highlights the importance of strengthening evidence-based health communication practices and promoting more balanced reporting that contextualizes both the potential benefits and the limitations of dietary supplement use.

Finally, the results of this study contribute to the literature by demonstrating how media framing shapes the presentation of dietary supplements within public information environments. By combining media analysis with a nutrition science perspective, this study provides insights into how health-related messages are constructed and communicated to the public. These findings may be relevant for nutrition professionals, policymakers, and science communicators seeking to improve the clarity, accuracy, and public health relevance of nutrition information disseminated through the media.

## 5 Conclusion

This study consists of a content and framing analysis of 45 news articles obtained through a simple random sampling of reports retrieved from the official website of the BBC, based in the United Kingdom, using the search terms *supplement*, *nutraceutical*, and *protein powder*. The findings derived from this analysis are interpreted within the broader framework of nutrition, food, and community health literature. The results provide important insights into how dietary supplements are represented in media discourse, particularly with regard to benefit–risk perceptions, scientific legitimacy, the influence of social media, and target-group–oriented patterns of use.

As a multidisciplinary study, the findings contribute to both Communication Studies and Nutrition Science. From a communication perspective, the content and framing analyses demonstrate that the news articles identified through the selected keywords are limited to a specific time period within the BBC’s searchable archive. In this respect, improving the accessibility and indexing of reports on dietary supplements—potentially through AI-supported search and categorization systems—may facilitate more comprehensive retrieval of related content. The simplified word list and context tables derived from the BBC news corpus also indicate strong thematic coherence within the sampled articles. The analysis reveals that five frames commonly expected in news coverage of dietary supplements—namely the Food Supplement Category Frame, Scientific Frame, Benefit–Risk Balance Frame, Social Media Frame, and Target Audience Frame—are present in the news texts. However, while most frames are clearly represented, the Social Media Frame appears relatively weak, despite the fact that a substantial share of contemporary supplement-related content circulates through social media platforms.

The contextual analysis further indicates that the news coverage generally adopts a relatively neutral stance from a scientific perspective. This neutrality is reflected in the balanced emphasis placed on both the benefits and risks of dietary supplements, often supported by references to expert opinions. Among the dietary supplements most frequently featured in the news are protein powders, collagen, vitamin D, magnesium supplements, taurine, and creatine. While some reports cite academic studies, expert opinions, and clinical or preclinical evidence, others provide limited or no scientific references. In this context, safety considerations and the balance between potential benefits and risks emerge as central evaluative criteria in media reporting. Cases describing adverse outcomes associated with misuse or excessive consumption further highlight the importance of appropriate dosage, individual characteristics, and safety considerations.

From a nutritional science perspective, these findings highlight the importance of nutritional literacy in shaping public understanding of dietary supplements. Media representations may influence perceptions of the necessity, safety, and effectiveness of supplements, thereby affecting consumption behaviors. Since decisions regarding supplement use are often influenced by family members, peers, magazines, and online sources rather than healthcare professionals, the quality and accuracy of nutrition-related information disseminated through the media become critically important for public health. Improving the clarity and scientific grounding of nutrition communication may therefore contribute to more informed decision-making and support the responsible use of dietary supplements.

The findings of this study also carry several practical and policy implications. For nutrition practitioners, the results underline the importance of actively participating in public communication processes and providing clear, evidence-based explanations regarding the appropriate role of dietary supplements within a balanced diet. For health journalists, the findings highlight the need for careful use of scientific sources and more comprehensive contextualization of supplement-related information in news reporting. Policymakers and public health institutions may also benefit from these insights by developing guidelines and communication strategies aimed at improving the accuracy and reliability of nutrition information in the media. Strengthening collaboration between nutrition scientists, media professionals, and regulatory bodies may help reduce misinformation and promote more responsible communication about dietary supplements.

Nevertheless, several limitations of this study should be acknowledged. The analysis was limited to selected media sources and a specific time period, which may not fully represent the broader media landscape. In addition, the study focused on media content rather than directly examining audience interpretation or behavioral outcomes. Future research could expand the scope by including different media platforms, such as social media channels and digital health influencers, and by investigating how media framing influences individuals’ perceptions, nutritional literacy, and supplement consumption behaviors. Expanding scientific research on the health effects of dietary supplements and developing harmonized regulatory approaches will remain important priorities for community nutrition and public health. Such research would contribute to a deeper understanding of the relationship between media communication, nutrition knowledge, and public health practices.

## Data Availability

The original contributions presented in the study are included in the article/supplementary material, further inquiries can be directed to the corresponding author.
